# The Roles of MicroRNAs in Male Infertility

**DOI:** 10.3390/ijms22062910

**Published:** 2021-03-13

**Authors:** Madalina Gabriela Barbu, Dana Claudia Thompson, Nicolae Suciu, Silviu Cristian Voinea, Dragos Cretoiu, Dragos Valentin Predescu

**Affiliations:** 1 Fetal Medicine Excellence Research Center, Alessandrescu-Rusescu National Institute for Mother and Child Health, 020395 Bucharest, Romania; mada.barbu93@gmail.com (M.G.B.); dana.lunganu@gmail.com (D.C.T.); dragos@cretoiu.ro (D.C.); 2 Department of Rehabilitation Medicine, Elias Emergency University Hospital, 011461 Bucharest, Romania; 3 Division of Obstetrics, Gynecology and Neonatology, Carol Davila University of Medicine and Pharmacy, 050474 Bucharest, Romania; 4 Department of Obstetrics and Gynecology, Polizu Clinical Hospital, Alessandrescu-Rusescu National Institute for Mother and Child Health, 011061 Bucharest, Romania; 5 Department of Surgical Oncology, Institute of Oncology Prof. Dr. Alexandru Trestioreanu, Carol Davila University of Medicine and Pharmacy, 022328 Bucharest, Romania; 6 Department of Cell, Molecular Biology and Histology, Carol Davila University of Medicine and Pharmacy, 050474 Bucharest, Romania; 7 Department of General Surgery, Sf. Maria Clinical Hospital, Carol Davila University of Medicine and Pharmacy, 011172 Bucharest, Romania; drpredescu@yahoo.com

**Keywords:** microRNAs, male infertility, azoospermia, reproductive dysfunctions

## Abstract

MicroRNAs applications were vastly studied throughout the years, spanning from potential cancer biomarkers to targeted therapies for various diseases. Out of these utilizations, this paper focuses on their role in male infertility. Approximately 10–15% of worldwide couples are affected by infertility. Out of these, 50% are due to male determinants. The majority of cases still have an undetermined cause. Previous studies have found that the aberrant expression of microRNAs could be linked to certain reproductive dysfunctions in males. Further on, this study looked into the most recent literature published on this subject in order to assess the connection between the up-/down-regulation of various microRNAs and the roles they play in male infertility. MicroRNAs were found to be abundant and stable in the seminal liquid, which led to a facile identification using regular RNA detection methods. It was observed that the concentration of microRNAs in semen was modified in the case of patients suffering from asthenozoospermia and azoospermia. Moreover, idiopathic male infertility was associated with a single nucleotide polymorphism of the microRNA binding site. Future studies should focus their attention on discovering future treatments against male infertility targeting specific microRNAs and also on developing new and improved contraceptive methods.

## 1. Introduction

Infertility is defined as the inability of a couple to obtain a spontaneous pregnancy after one year of regular, unprotected sexual intercourse [1]. Worldwide, about 15% of couples are affected by conditions that lead to infertility, out of which up to 50% are believed to be due to the male determinant [2,3]. According to one study, globally, over 30 million males suffer from infertility [2]. There are many known causes linked to male infertility, amongst them being various congenital or acquired urogenital dysfunctions, hormonal dysregulation, urogenital tract infections, genetic mutations and scrotal hyperthermia that can occur in varicocele, prolonged vehicle driving or laptop users that keep their laptop computers on their thighs for long periods of time [4,5,6]. Thus, reproductive inability can be attributed to congenital, genetic or environmental factors, yet up to 60–75% of diagnosed male infertility was found to be idiopathic [6].

Some studies suggested that small molecules of non-coding RNA, known as microRNAs, might also be involved in male infertility [7]. microRNAs can be found in all eukaryotic cells and were proved to modulate various physiological processes through gene up- or down-regulation [7]. The dysregulation of these molecules was linked to the development of numerous diseases such as cancers, neurodegenerative disorders and viral infections, thus generating the need for extensive research to be made into the possibility of using them as diagnostic biomarkers or for targeted therapies in the above-mentioned pathologies [8,9,10,11]. Present in almost all tissues and body fluids, numerous microRNAs were also isolated in semen samples, their modified expression levels being associated with reduced sperm count, low sperm motility and abnormal sperm morphology [12,13,14,15].

The latest studies discovered that while some transcribed RNA molecules encoded proteins during genome transcription, the majority of them were non-coding [16,17]. What was previously considered to be “junk” RNA has been recently proved to have important regulatory functions in gene expression during the transcriptional and post-transcriptional phases [16]. microRNAs, however, generally exert their functions at a post-transcriptional level, thus influencing the translation or stability of the targeted messenger RNA (mRNA) [18].

**Figure 1:**
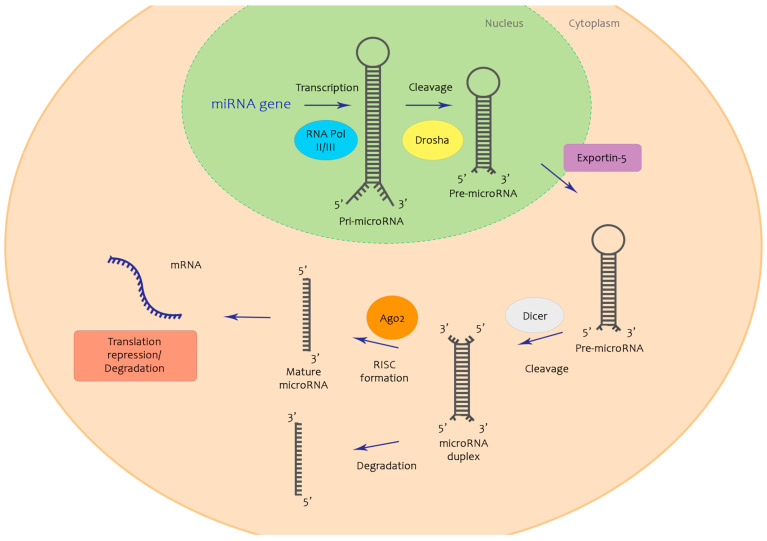
Exemplification of microRNA biogenesis. MicroRNA genes hold the genetic information necessary for the transcription of the pri-microRNA, via RNA polymerase II and III. This is further cleaved by Drosha enzyme to pre-microRNA, which is transported to the cytosol by Exportin-5. Dicer enzyme cleaves the molecule one more time, forming a microRNA duplex from which will emerge two separate strands—the passenger strand, often degraded, and the guide strand, which will form the RNA-induced silencing complex (miRISC). This will exert its role on mRNA, repressing its translation.

microRNA genes located in intragenic and intergenic regions are transcribed by RNA polymerase II and III, resulting in a hairpin-like molecule which is thereafter processed first by a RNase III enzyme known as Drosha which shortens it to approximately 70 nucleotides, representing the precursor-microRNA (pre-microRNA) [19,20,21,22]. This precursor is further transported by Exportin-5 to the cytoplasmatic space where it is once again cleaved by an RNase III enzyme known as the Ago2/Dicer complex, giving rise to small, mature double strands of microRNA [23]. Afterward, the passenger strand will be most often degraded, while the guide strand will form the RNA-induced silencing complex (miRISC) through which microRNAs exert their gene regulatory functions by binding to their targeted mRNA [24] (Figure 1). According to previous literature, one microRNA can target multiple mRNAs and, in turn, one mRNA could possess binding sites for numerous microRNAs [25].

## 2. MicroRNA Involvement in Spermatogenesis

Spermatogenesis is defined as the differentiation process of germ cells (GC) into mature male gametes, known as spermatozoa [26]. The process takes place entirely in the seminiferous tubules of the male gonads and comprises spermatogonia proliferation, differentiation of spermatogonia into spermatocytes, which further enter meiotic division, resulting in the development of spermatids; the latter undergo maturation, giving rise to highly specialized spermatozoa [27]. Furthermore, spermatogonia are also able to enter a mitotic process in order to renew themselves, thus maintaining the germ-cell pool constant throughout life [27]. Any disturbance during the stages of spermatogenesis could lead to impaired male fertility.

Sertoli cells, which are located in the epithelium of seminiferous tubules, were proved to be indispensable for spermatogenesis. With a dynamic morphology, their tree-branch-like ramifications support and assist for migration of numerous germ cells [27,28]. They secrete numerous factors, such as androgen-binding protein, transferrin, growth factors and interleukins, which ensure a favorable microenvironment for the maturation of gametes [29,30]. Furthermore, through the secretion of a hormone known as inhibin-B, Sertoli cells are able to modulate the synthesis and secretion of follicle-stimulating hormone (FSH) through a negative feedback loop [27]. Studies showed that Sertoli cells were also able to function as macrophages in order to remove degenerated germ cells [27]. 

The activity of Sertoli cells is highly regulated by the hypothalamic–pituitary–gonadal axis, through the secretion of FSH, which appeared to be involved in numerous processes regarding spermatogenesis. However, studies showed that the absence of FSH or a mutation in its receptor, although impairing spermatogenesis, does not abolish it [27]. While FSH levels have an impact on the activity of Sertoli cells, another hormone of the hypothalamic–pituitary–gonadal axis, known as the luteinizing hormone (LH), exerts its actions on Leydig cells, testicular cells that are responsible for testosterone and estrogen synthesis and secretion, both of them being vital for normal spermatogenesis [31,32].

The complexity of spermatogenesis resides in the myriad of events involved in this process that are regulated at a genetic, cellular and epigenetic level and in which hormones play a central role. From a genetic standpoint, a number of transcription factors have been proven to play an important part in the development and maturation of spermatozoa, some of which are expressed in germ cells (heat shock factor 2—HSF2 and OVOL1), Sertoli cells (RHOX5, WT1, SOX8) and epididymis (FOXI1), where they exert various key roles [33]. Furthermore, at the cellular level, metabolic regulation has also been proven to affect spermatogenesis. For instance, Sertoli cells produce lactate from glucose, which provides the necessary energy substrate for the germ cells and also prevents their apoptosis [34]. However, more recent animal studies have shown that microRNAs could also have an impact on spermatogenesis. While it was well known that these small non-coding molecules of RNA were responsible for the modulation of numerous physiological processes, only in recent years research started focusing on determining the role they play in spermatogenesis and how their dysregulation may affect fertility. Determining their patterns of expression in the testicular tissue may lead to the development of new diagnostic or treatment tools that could lower the impact of infertility on couples worldwide.

Previous studies have identified several microRNAs located in the testis and, in particular, testicular cells such as different types of germ cells and Sertoli cells [26]. Numerous specific microRNAs were discovered in spermatogonial stem cells (SSCs), with roles ranging from the initiation or blockage of the differentiation process to the modulation of the self-renewal mechanisms (Table 1). Studies conducted on mice showed that miR-146 presented higher expression levels in undifferentiated spermatogonial cells, being associated with the activity of the retinoic acid signaling pathway that plays a crucial role in both the initiation of the differentiation process and the passing into the meiosis state of male germ cells [35]. miR-146 was observed to directly bind and inhibit a coregulator of retinoid receptors known as the mediator complex subunit 1 (Med1) [35]. High levels of retinoic acid were found to inhibit the expression of miR-146 in undifferentiated spermatogonia, while the up-regulation of miR-146 was considered to be sufficient to antagonize the impact that retinoic acid has on spermatogonia [35].

Pertaining to the role miR-34b/c plays in spermatogenesis, studies were divided. Some reported that both miR-34b/c and miR-449a/b/c were crucial for the first cleavage division in in vitro studies [36], while others showed that miR-34b/c and miR-449-null test animals presented a normal spermatogenesis [37]. An interesting fact is that these microRNAs, which belong to the same microRNA family due to having an identical “seed sequence”, were only isolated in spermatozoa, not in oocytes, making them paternal microRNAs [36,37]. The previously described study concluded that although not essential for male fertility, the absence of both miR-34b/c and miR-449 would result in disrupted spermatogenesis and male infertility due to abnormal chromatin condensation and spermiogenic disruptions [37]. Furthermore, miR-34c was also found to induce the apoptosis of germ cells by targeting the activating transcription factor 1 (ATF1) gene, expressed by spermatocytes [38]. High levels of miR-34c were also detected in spermatocytes and round spermatids, where its inhibition was also found to decrease germ-cell apoptosis [26].

There were several testis-specific microRNAs identified, amongst them being miR-202-3p and miR-202-5p, also found in high levels in undifferentiated spermatogonia of mouse models [39]. While having similar expression levels in SSCs, this trend changed in other testicular cells, such as Leydig and Sertoli cells, where higher levels of miR-202-5p were detected [39]. The glial cell-derived neurotrophic factor (GDNF), a known facilitator of SSCs self-renewal, was found to increase the expression of miR-202-3p, while retinoic acid acted as a down-regulator [39]. In contrast, it was proved that retinoic acid increased the expression of miR-202-5p [39]. Furthermore, the in vitro inhibition of miR-202-3p led to the initiation of differentiation and decreased by 75% the activity of stem cells, thus suggesting that one of the roles of miR-202-3p would be to maintain spermatogonia in an undifferentiated state [39]. In miR-202 knockout models, an accelerated cell cycle of SSCs and an increase in the spermatogonia apoptosis were observed [39]. 

Several microRNAs were detected in Sertoli and Leydig cells as well. One study identified three testis-specific microRNAs like miR-471, miR-463 and miR-201, located on the X-chromosome that were supposed to interfere with the androgen activation of Sertoli cells [40]. The most studied was miR-471, which seemed to target and modulate the activity of both Dsc1 (desmocollin 1) and Foxd1 (forkhead/winged-helix transcription factor) [40]. The first one is thought to be involved in the proper functioning of the blood–testis barrier, while Foxd1 is involved in post-meiotic germ-cells maturation and development [40]. Although further studies are needed in order to truly understand the mechanisms through which microRNAs regulate the activity of Sertoli cells, it can be stated that they are essential for a good coordination of some Sertoli cell processes.

As previously stated, the main function of Leydig cells is to synthesize steroid hormones, the most important one being testosterone. It was shown that a disruption in the activity of Leydig cells associated with decreased testosterone production would result in aspermatogenesis [41]. While most studies were conducted on spermatogonial stem cells or Sertoli cells, very few were focused on examining the roles of Leydig cells in the modulation of spermatogenesis via microRNAs. One recent study conducted on mice showed that miR-150, which was found to be predominantly expressed in Leydig cells, is a down-regulator of STAR, a protein involved in the transfer of cholesterol into the mitochondria during steroidogenesis [42]. While the paper showed that the miR-150-STAR pathway could play a crucial role in both steroid synthesis and spermatogenesis, further studies are required in order to discover the in vivo mechanisms through which microRNAs exert their functions in Leydig cells.

**Table 1:** Overview of the most studied microRNAs involved in spermatogenesis.

microRNA	Location	Action	Type of Sample	Study Population	References
miR-34c	SSCs, spermatocytes and round spermatids	Induce apoptosis Up-regulation of GC-specific genes Paternal microRNAs	Epididymal sperm [36], primary spermatocytes [37], adult mouse testis [38]	Mice	[36,37,38,43]
miR-449		Induce apoptosis Up-regulation of GC-specific genes Paternal microRNAs	Epididymal sperm [36], primary spermatocytes [37], adult mouse testis [38]		[36,37,38,43]
miR-21	SSCs	When down-regulated—increased apoptosis and decreased potency of SSCs Seems to be involved in SSCs self-renewal	Germ-cell cultures	Mice	[44]
miR-146a	SSCs	Regulates the differentiation process by binding to Med1; overexpression seems to block the retinoic acid-induced differentiation of SSCs	Germ-cell cultures	Mice	[35]
miR-20	SSCs	Promotor of SSCs self-renewal, by targeting STAT3 and Ccnd1	Germ-cell cultures	Mice	[45]
miR-106a	SSCs	Promotor of SSCs self-renewal, by targeting STAT3 and Ccnd1	Germ-cell cultures	Mice	[45]
miR-202-3p	SSCs	Up-regulated in mice SSCs Up-regulated by GDNF and downregulated by RA Its absence leads to premature differentiation, decreased activity of stem cells, mitosis and apoptosis Testis-specific	Germ-cell cultures	Mice	[39,46,47]
miR-202-5p	SSCs	Up-regulated by RA Testis-specific	Germ-cell cultures	Mice	[39]
miR-471	Sertoli cells	Testis-specific Regulates the expression of FoxD1 and Dsc1 Blood-testis barrier Post-meiotic germ-cell maturation	Isolated Sertoli cells from mice testis	Mice	[40]
miR-463	Sertoli cells	Testis-specific	Isolated Sertoli cells from mice testis	Mice	[40]
miR-201	Sertoli cells	Testis-specific	Isolated Sertoli cells from mice testis	Mice	[40]

## 3. The Implications of MicroRNA Dysregulation on Male Infertility

Seeing how numerous microRNAs are involved in all steps of spermatogenesis, it is only logical that their dysregulation would cause various male infertility-related issues. Modified levels of microRNAs were already proved to be associated with the development of multiple diseases, such as various types of cancer, viral infections and neurodegenerative disorders, and recent studies focused on their involvement in infertility [7,8,9,10,11]. It is believed that some of the numerous cases of male infertility that were previously ruled as idiopathic may be explained by changes in the expression of particular microRNAs involved in the process of spermatogenesis.

Studies showed that a disruption in the synthesis of microRNAs, such as deletions in the Dicer gene (Dicer1), greatly impacted spermatogenesis [48]. For instance, when occurring in the epididymal cells of mice, it was observed that it impaired the maturation of germ cells, while when located in Sertoli cells, spermatozoa were entirely missing, together with the development of testicular degeneration [49,50]. Furthermore, deletions in the Dicer gene in male germ cells led to impaired differentiation of haploid spermatids, followed by apoptosis and failure of spermatogenesis in the haploid and meiotic stages [51,52,53]. In Dicer1 knock-out mice, the absence of the Dicer gene was not only associated with diminished spermatogenesis, but also with an inability of sperm cells to fertilize the oocyte in vitro [48,49]. 

One of the most studied microRNAs pertaining to male infertility is miR-34. Recently, a study was published comparing a total of 106 semen samples, out of which 40 were declared to have normozoospermia, 47 had asthenozoospermia and 19 oligozoospermia [54]. The results showed that miR-34b-5p, as well as miR-34c-3p and miR-34b-3p, were significantly less expressed in the semen samples of patients with oligozoospermia than in those with normozoospermia [54]. Furthermore, numerous studies found that reduced levels of miR-34b and miR-34c were associated with decreased male fertility, non-obstructive azoospermia, defective meiosis and spermatozoa maturation and spermatogenic disruptions [37,55,56,57,58,59]. Similar to these findings, miR-449 dysregulation was also linked to male infertility, due to impaired motility of the spermatozoa [59].

When comparing the semen plasma samples of fertile men with those of infertile men of the same age group, in a large study on 457 patients, Wang C., et al. detected seven microRNAs that were significantly down-regulated in the samples of patients with azoospermia, while they were up-regulated in samples of patients with asthenozoospermia [60]. These microRNAs were miR-34c-5p, which was already mentioned above, and miR-122, miR-146b-5p, miR-181a, miR-374b, miR-509-5p and miR-513a-5p [60]. Another study conducted on 192 patients suffering from idiopathic male infertility showed that miR-19b and let-7a were significantly increased in the seminal plasma samples of patients suffering from non-obstructive azoospermia, compared to control samples from fertile men [61]. All these microRNAs could potentially be used as diagnostic biomarkers for idiopathic male infertility, as well as in the development of innovative targeted therapeutic tools.

## 4. The Potential of MicroRNAs as Biomarkers

Numerous studies around the world have demonstrated the potential role microRNAs could play as biomarkers. This key function has been assessed for diseases such as cancer [62,63], cardiovascular disease [64,65,66,67], neurological conditions [68,69,70,71,72,73], viral infections [74] and many others. In the field of in vitro fertilization, there are still multiple questions that need to be answered regarding the cause of male infertility, and better diagnosis techniques need to be developed in order for improved treatments to have the possibility to emerge. One of these techniques involves the use of various microRNAs as potential biomarkers.

Previous research has shown that in up to 20% of the cases, male infertility is responsible alone for the inability to conceive, while in a further 30–40% of cases, it acts as a contributor [75]. Many of the conventional tests available today for the diagnosis of unexplained male infertility, including the histological assessment of the testicular tissue, are lacking both in sensitivity and specificity to be able to provide an accurate diagnosis [76,77,78,79]. All of the above-mentioned, together with a lack of findings during the physical examination and also a normal anamnesis, lead to a diagnosis of idiopathic male infertility in up to 75% of the cases [56]. MicroRNAs have the potential to answer some of these problems. Various genomic analyses have indicated that the dysregulation of cell- or germ-specific microRNAs could be responsible for this type of pathology in a number of spermatogenic impairments, such as oligozoospermia, asthenozoospermia and oligoasthenozoospermia, and also in some histopathological patterns in the testes (germ-cell arrest, Sertoli cell only, mixed atrophy) [60,76,80,81,82,83].

The main characteristics of a good biomarker are represented by a high specificity and sensitivity, their potential to be non-invasive and their easily accessible source. Even though most of the biomarkers are nowadays represented by proteins, the circulating microRNAs constitute good potential candidates for this role in male infertility, as they are abundant in the seminal plasma, serum and plasma of the patients, can be easily detected even at low concentrations and could be used as a non-invasive method of diagnosis and treatments of such cases [56,83]. Moreover, microRNAs are also more homogenous than protein biomarkers and are more stable than RNA molecules, even at room temperature and after multiple freeze–thaw cycles [60,63,84,85]. Some estimates suggest that up to 30% of the genes that encode proteins are regulated by microRNAs, and over 60% are regulated by an array of microRNAs [86].

Several studies in the past have looked upon the use of these molecules as potential diagnosis biomarkers. According to one of these papers, the levels of miR-122, miR-185, miR-574-5p, miR-297, miR-373, miR-1275 and miR-193b were up-regulated in the whole semen of patients suffering from infertility, while the levels of miR-16, miR-100, miR-19b, miR-512-3p, miR-26a and miR-23b were found to be under the normal values [87]. Reduced levels of miR-10b and miR-135b were also found in the case of males suffering from asthenospermia, while the ones that were suffering from oligoasthenospermia had miR-34c5, miR-181a and miR-122 significantly down-regulated [88,89]. Many more such published works have shed light on various microRNAs that could be used as potential biomarkers for the diagnosis of male infertility [56,90,91,92]. Even though the use of proteins as biomarkers may not be completely replaced by these molecules, their high efficiency and early-detection possibilities make them good candidates for complementary diagnosis tools.

However, microRNAs also have their drawbacks. One of their disadvantages is represented by the recognition of potential contenders to be used as biomarkers. This is a strenuous process, currently performed using mathematical algorithms through the use of bioinformatics. The wide variety of existent software often turns finding a correct microRNA target into a controversial and exhaustive task. Furthermore, the fact that one single microRNA could be responsible for the regulation of several genes or that multiple microRNAs could be linked to the same gene makes this task even more difficult for researchers. One of the solutions proposed for this matter was the use of microRNA panels instead of isolated microRNA molecules [93,94]. Using this model, several drawbacks could be avoided, such as the case of multiple microRNAs acting upon the same target gene [95,96], the fact that these molecules tend to stay in clusters thus making it difficult to assess the sequence responsible for the actual binding [97] and also the seed regions similarities amongst members of the same family that make them hard to differentiate [98]. Other disadvantages for the use of microRNAs as potential biomarkers in male infertility are represented by the lack of universal protocols for the collection, storage and processing of the samples and by the fact that there is a missing validation of the obtained data. Further collaborative work needs to be done in order for these molecules to reach a routine clinical use.

## 5. Perspectives

Besides their potential use as biomarkers, microRNAs have also been considered for therapeutic uses as part of the personalized medicine field involving male infertility. Researchers are studying various ways to alter their levels, either by supplementing them with microRNA mimics in the case of down-regulation [99] or by inhibiting their expression using laboratory-designed molecules called anti-miRs for the situations when they are over-expressed [100]. A recent study showed how a microRNA mimic (a fabricated oligonucleotide) called MRX34, designed to imitate miR-34a, which was integrated in a lipid capsule, managed to suppress the progression of a tumor [101]. Opposed to this, anti-miRs exert their inhibitory function through binding to a predetermined microRNA, thus blocking it. Both of these therapies have been previously tested for a variety of cancers or viral infections but never for the purpose of treating male infertility [45,102].

Moreover, studies have also shown that microRNAs play a crucial role in the development of testicular germ-cell tumors in males suffering from infertility in comparison to fertile men [103,104]. Similar to other types of cancer, microRNAs have the potential to act both as oncogenes, or tumor suppressors, opening the possibilities for future therapeutic targets [105,106,107,108,109,110]. For example, the miR-371-373 cluster has been found to play the role of an oncogene in the development of testicular germ-cell cancer [111]. The probable mechanism involves the inhibition of large tumor suppressor kinase 2 (LATS2), leading to a further blockage of the p53 tumor protein signaling pathway [111]. Furthermore, another study showed that some microRNAs could be used to discriminate between type II and type III testicular germ-cell tumors and also to differentiate the histological parts in type II [112]. Depending on the effect they have in the development of cancer, two models were proposed for microRNAs to be used in the targeted modulation therapies. On one hand would be the suppression of the potential oncogenes, while on the other would be the promotion of those displaying a tumor-suppressing function [113]. However, in order to achieve this desiderate, there are a number of issues that need to be overcome. Some of these include, but are not limited to, the high costs of modified nucleic acids and the development of a more targeted delivery method as these molecules exert different functions in various tissues.

## 6. Conclusions

Idiopathic male infertility is still one of the great challenges modern medicine faces in its pathway to improve in vitro fertilization techniques, and understanding it is crucial for offering the couples in need of it a chance to have a baby. Moreover, the links between infertility and cancer are becoming increasingly evident with the development of improved diagnosis techniques. MicroRNAs have been shown over the past years to influence numerous biological processes, spermatogenesis being one of them, together with testicular tumorigenesis. Their dysregulation could prove to be the key in the development of new and enhanced diagnostic and prognostic techniques for infertile men and could also offer new treatment possibilities in the field of personalized medicine. For their potential to be fully exploited more research is needed in order to discover the types of microRNAs involved in these processes, the mechanism through which they act and how the modulation of their expression could change the outcome for these patients.
